# Hybrid Films Based on Bilayer Graphene and Single-Walled Carbon Nanotubes: Simulation of Atomic Structure and Study of Electrically Conductive Properties

**DOI:** 10.3390/nano11081934

**Published:** 2021-07-27

**Authors:** Michael M. Slepchenkov, Pavel V. Barkov, Olga E. Glukhova

**Affiliations:** 1 Institute of Physics, Saratov State University, 410012 Saratov, Russia; slepchenkovm@mail.ru (M.M.S.); barkovssu@mail.ru (P.V.B.); 2 Laboratory of Biomedical Nanotechnology, I.M. Sechenov First Moscow State Medical University, 119991 Moscow, Russia

**Keywords:** hybrid films, single-walled carbon nanotubes, graphene nanoribbons, electron transmission function, electrical resistance, charge transfer, local stress, energy stability

## Abstract

One of the urgent problems of materials science is the search for the optimal combination of graphene modifications and carbon nanotubes (CNTs) for the formation of layered hybrid material with specified physical properties. High electrical conductivity and stability are one of the main optimality criteria for a graphene/CNT hybrid structure. This paper presents results of a theoretical and computational study of the peculiarities of the atomic structure and the regularities of current flow in hybrid films based on single-walled carbon nanotubes (SWCNTs) with a diameter of 1.2 nm and bilayer zigzag graphene nanoribbons, where the layers are shifted relative to the other. It is found that the maximum stresses on atoms of hybrid film do not exceed ~0.46 GPa for all considered topological models. It is shown that the electrical conductivity anisotropy takes place in graphene/SWCNT hybrid films at a graphene nanoribbon width of 4 hexagons. In the direction along the extended edge of the graphene nanoribbon, the electrical resistance of graphene/SWCNT hybrid film reaches ~125 kOhm; in the direction along the nanotube axis, the electrical resistance is about 16 kOhm. The prospects for the use of graphene/SWCNT hybrid films in electronics are predicted based on the obtained results.

## 1. Introduction

In the last decade, the possibility of combining graphene and CNTs has attracted much attention from the research community in order to obtain new multifunctional hybrid materials with promising properties due to the synergistic effect caused by the interaction of nanostructures of various dimensions—1D nanotubes and 2D graphene [1,2,3,4,5,6,7,8,9,10]. In addition, hybridization of graphene and CNTs will make it possible to avoid the agglomeration of individual CNTs and graphene sheets, which leads to a significant deterioration in the properties of the aforementioned carbon nanomaterials in a real experiment. Experimenters have proposed various approaches to the formation of hybrid nanostructures based on graphene and CNTs, which differ in both the manufacturing technology and the type of topological architecture of the created hybrids [11,12,13,14,15]. There are three most general topological types of hybrid graphene/CNT nanostructures: (1) horizontally-oriented CNTs are located in the graphene plane; (2) vertically oriented CNTs are located perpendicular to the graphene plane; (3) CNTs “wrapped” with graphene sheets [16]. The most common is the first of the topological types described above. Both SWCNTs and multi-walled CNTs (MWCNTs) are used for its implementation: they can either cover graphene or act as a substrate for it [17,18,19,20,21,22]. From the standpoint of practical application, it is preferable to use SWCNTs for the creation of graphene/CNT hybrid structures since SWCNTs have better properties as compared to MWCNTs, including a higher specific surface area, a lower defect density, and tunable electronic characteristics in accordance with the chirality of nanotubes, which are especially important for nanoelectronic devices.

Graphene/SWCNT hybrid structures with horizontally oriented nanotubes find their application as a material for creating the element base of various nanodevices. In particular, highly sensitive photodetectors have been developed on the basis of atomically thin graphene/SWCNT hybrid films. A photodetector based on van der Waals graphene/SWCNT heterostructures located on a SiO_2_/Si substrate has been presented by Liu et al. [23]. The proposed photodetector showed a significant increase in photoconductivity (~10^5^), fast response time (~100 μs), and ultra-wideband sensitivity in the range of electromagnetic radiation from visible to near-infrared. Liu et al. have also developed the flexible photodetector utilizing van der Waals bonded graphene-SWNT hybrid films as the light-harvesting layer. This device on a flexible polyethylene terephthalate substrate demonstrated a high photoresponsivity of ~51 A/W and a fast response time of ~40 ms over the visible wavelength range [24]. An all-carbon hybrid humidity sensor based on graphene/semiconducting SWCNT van der Waals heterostructures has been developed by Cai et al. This sensor demonstrated good reproducibility and high sensitivity of 24% per 1% relative humidity change over a wide range of relative humidity (5 to 80%), as well as a short response/recovery time of 198/110 ms [25]. A hybrid architecture consisting of SWCNT films coated with platinum and graphene particles has been successfully used as an electrode for detecting H_2_ molecules. It was found that the H_2_ response in SWCNT/graphene nanohybrid improved by 50% as graphene provides more efficient charge transfer [26]. Layered hybrid structures based on polymerized SWCNTs and graphene are promising materials for anode electrode in lithium-ion batteries. It was shown that such structures have a high specific capacity of ~630 mA∙h∙g^−1^, outstanding power capability of ~390 mA∙g^−1^, and cyclability over 1000 operating cycles with maximum Coulombic efficiency [27]. Composite layered structures of graphene and SWCNTs have shown no fewer promising results in the development of supercapacitors with ultrahigh energy density. Electrodes based on graphene/SWCNT layered films showed an ultra-high energy density of 155.6 Wh∙kg^−1^ in an ionic liquid at room temperature. In addition, an increase in the specific capacity of electrodes by 29% after 1000 operating cycles were recorded, which indicates their excellent cyclicity [28].

Along with the development of various devices based on graphene/SWCNT hybrid structures, fundamental studies of the regularities of physical processes in these structures are also being carried out. The mechanisms of thermal and electrical conductivity in graphene/SWCNT hybrid structures are studied using computer simulation methods. Using the method of nonequilibrium molecular dynamics, it was found that the thermal boundary conductivity in hybrid structures based on horizontally oriented SWCNTs and graphene nanoribbons increases linearly with increasing temperature due to the significant role of inelastic phonon scattering. It was shown that as the strength of the interphase interaction increases, the thermal boundary conductivity monotonically increases due to the emerging enhanced phonon coupling between SWCNTs and graphene [29]. Based on the results of DFT calculations performed for an atomistic model comprising one armchair and two zigzag semiconducting nanotubes, it was shown that the addition of graphene flakes introduced additional electronic states at the Fermi level, which increased the current and conductivity of graphene/SWCNT hybrid system [30]. In turn, it was experimentally revealed that when a SWCNT film is deposited on a graphene substrate, its conductivity increases by approximately the same amount as in chemical doping due to a decrease in the height of the tunnel barrier inside the carbon material [31]. At the same time, the issues of controlling the conductive properties of graphene/SWCNT hybrid structures by choosing a certain topology of nanotubes and graphene, as well as their mutual orientation in the hybrid architecture, remain poorly understood. Taking into account the possibilities of modern experimental technologies, including the controlled growth of SWCNTs of a certain chirality, the transfer of SWCNT films onto a graphene substrate of atomic thickness, as well as the coating of SWCNT films with a thin graphene layer, it can be assumed that it is the topological control of the properties of graphene/SWCNT hybrid structures that will serve as a key for further development of the elemental base of nanodevices on an industrial scale.

The aim of this paper is to study the peculiarities of the atomic structure and the regularities of current flow in hybrid films formed by bilayer zigzag graphene nanoribbons and a SWCNT (12,6) layer using theoretical and computational methods. The choice of SWCNTs (12,6) and zigzag graphene nanoribbons is due to the following reasons. Chiral SWCNTs (12,6) with a diameter of 1.2 nm are among the most frequently synthesized single-walled nanotubes [32,33,34]. In addition, the SWCNT (12,6) has a metallic type of conductivity, which indicates its prospects for use as electrodes and connecting conductors in electrical circuits, as well as for the manufacture of transparent conductive films in flexible electronics devices [35,36]. The choice of graphene nanoribbons with a zigzag edge is due to their unique transport properties, in particular, the presence of localized edge states with energies close to the Fermi level [37], which predetermines their broad prospects for use in nanoelectronic devices, in particular in spintronic devices [38,39].

## 2. Computational Details

The atomic structure of graphene/SWCNT hybrid films was calculated using the self-consistent charge density-functional tight-binding (SCC-DFTB) method [40]. This method has been thoroughly tested in the study of carbon nanostructures of various topologies [41]. The SCC-DFTB method allows one to study the electronic structure, energy, and distribution of the density of electronic states for polyatomic supercells containing several hundred atoms and even thousands of atoms, which cannot be realized by the DFT method.

In order to quantitatively evaluate the stability of graphene/SWCNT hybrid films to internal deformations, the distribution of local stresses of the atomic network was calculated. The stress on each atom was calculated as the ratio of the force acting on the atom to the surface area of the atom, taking into account its immediate environment. The force was defined as the derivative of the energy of the atom with respect to its coordinates x, y, z. The energy of each atom was calculated by the SCC-DFTB method in the DFTB+ software package version 20.2 [42,43].

The electrical conductivity was calculated within Landauer–Buttiker formalism [44]. This formalism allows calculating the electron transmission function *T*(*E*) and static electrical conductivity *G*. The function *T*(*E*) is determined by the expression:

(1) $$T\left(E\right)=\frac{1}{N}\sum_{k=1}^{N}\mathrm{Tr}\left(\Gamma_{S}\left(E\right)G_{C}^{A}\left(E\right)\Gamma_{D}\left(E\right)G_{C}^{R}\left(E\right)\right),$$

where $G_{C}^{A}\left(E\right)$, $G_{C}^{R}\left(E\right)$ are advanced and retarded green matrices describing the contact with the electrodes, $\Gamma_{S}\left(E\right)$, $\Gamma_{D}\left(E\right)$ are the level broadening matrices for the source and drain. Static conductivity is described by the expression:

(2) $$G=\frac{I}{V}=\frac{2e^{2}}{h}\int_{-\infty }^{\infty }T\left(E\right)F_{T}\left(E-E_{F}\right)dE,$$

where *E_F_* is the Fermi energy of the material of the contacts, *F_T_* is the thermal broadening function; *e* is the electron charge, *h* is the Planck’s constant, *e*^2^/*h* is the quantum of conduction (this value is doubled to account for the electron spin).

In this study, the calculations of the transmission function *T*(*E*) were carried out for supercells of graphene/SWCNT hybrid films consisting of 250–300 atoms. In view of the large dimension of the considered electronic system, the original method for the accelerated calculation of the transmission function *T*(*E*) was applied using the original Mizar program [45]. This method allows to calculate *T*(*E*) for a small number of *k*-points of the first Brillouin zone and then interpolating it for any *k*-point of the first Brillouin zone and recovering the full transmission function *T*(*E*).

To analyze the electronic distribution in the studied graphene/SWCNT hybrid films, the Mulliken Population Analysis (MPA) was carried out [46]. According to Mulliken, the electron density of an atom is determined by the sum of the squares of the coefficients of the expansion of molecular orbitals into atomic orbitals of a given atom plus half of the cloud of overlapping orbitals of a given atom with a neighboring atom.

## 3. Results

### 3.1. Atomic Structure of Graphene/SWCNT Hybrid Films

**Figure 1:**
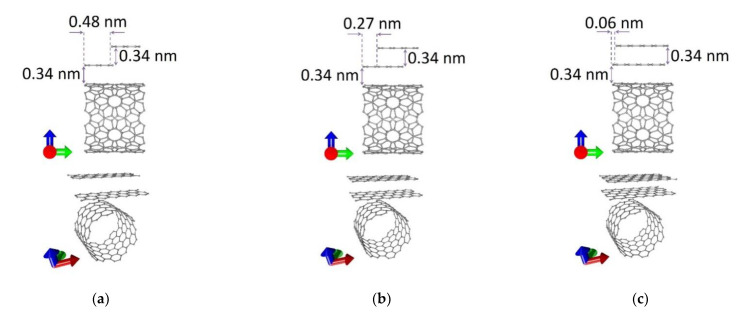
Initial atomic structure of supercells of graphene/SWCNT hybrid films: (**a**) model V1; (**b**) model V2; (**c**) model V3.

To construct atomistic models of graphene/SWCNT hybrid films, we used SWCNTs (12,6) with a diameter of 1.2 nm and bilayer zigzag graphene nanoribbons. One of the graphene layers was shifted relative to the other in the direction of the Y-axis (along the nanotube axis). Wherein bilayer graphene was located above the SWCNT surface at a distance of 0.34 nm. In total, three topological models of the graphene/SWCNT hybrid films were considered. These models differed in the width of the graphene nanoribbon in each layer. With an increase in the width of the nanoribbon, the displacement of the graphene layers relative to each other in the direction of the Y-axis (along the nanotube axis) changed. In the first version (model V1) of the initial graphene/SWCNT film, the width of the graphene nanoribbon was 2 hexagons (~0.5 nm), the displacement of the layers along the Y-axis was 0.48 nm. In the second version (model V2), the width of the graphene nanoribbon was 3 hexagons (0.71 nm), the displacement of the layers was 0.27 nm. In the third version (model V3), the width of the graphene nanoribbon was 4 hexagons (0.92 nm), the displacement of the layers was 0.06 nm. The distance between graphene layers along the Z-axis (perpendicular to the nanotube axis) was 0.34 nm for all considered topological models of graphene/SWCNT hybrid films. Figure 1 shows the initial atomic structures of the supercells of graphene/SWCNT hybrid films. The translation vectors of the initial supercells of graphene/SWCNT structures in the direction of the X-axis (along the extended edge of the graphene nanoribbon) and Y-axis (along the nanotube axis) were *L_x_* = 1.72 nm and *L_y_* = 1.12 nm, respectively.

**Figure 2:**
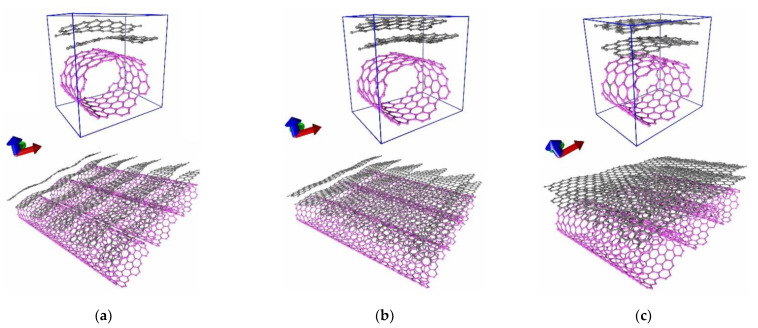
Atomistic models of supercells and extended fragments of graphene/SWCNT hybrid films: (**a**) model V1; (**b**) model V2; (**c**) model V3.

The initial atomic structures of the supercells were optimized to find their equilibrium configuration, i.e., configurations characterized by the minimum energy. The atomic structure of the supercells was optimized by the SCC-DFTB method. In the course of optimization, a relaxation scanning of the energy surface of the hybrid film was carried out. This scanning implied a step-by-step change in the lengths of the translation vectors *L_x_*, *L_y_* with the optimization of the atomic network geometry of the supercell constructed for these *L_x_*, *L_y_*. The lengths of the translation vectors of the supercells after optimization are shown in Table 1. Figure 2 shows the equilibrium configurations of the constructed supercells of graphene/SWCNT hybrid film. This figure also shows extended fragments obtained by translating each of the supercells in two directions (along the X and Y axes). It can be seen that after finding the equilibrium configuration, the atomic structure of both nanotubes and graphene nanoribbons underwent significant changes. The nanotube was deformed due to its attraction to the graphene layers, changing the ratio between the radii in the direction of the X and Y axes (*r_x_* and *r_y_*, respectively). Table 1 shows the calculation data for the degree of deformation (*r_x_*/*r_y_*) of nanotubes in graphene/SWCNT hybrid structure. According to these data, the degree of deformation was 1.2 for all three topological models. The atomic structure of the initial graphene nanoribbons was also deformed as a result of the interaction of nanoribbons with each other and with the nanotube. Moreover, depending on the width of the graphene nanoribbon, different orientations of the graphene layers with respect to the nanotube were observed. As can be seen from Figure 2a,b, supercells with nanoribbon widths of 2 and 3 hexagons, as a result of translation in two directions, formed extended 2D graphene/SWCNT structures, in which graphene nanoribbons were located at an acute angle with respect to the nanotube surface, resembling a “comb”-type structure. As can be seen from Figure 2c, a supercell with a nanoribbon width of 4 hexagons, as a result of translation in two directions, formed an extended 2D graphene/SWCNT structure, where graphene nanoribbons transformed into bilayer graphene sheets covering the nanotubes. That is, with a width of 4 hexagons, the translated fragments of graphene nanoribbons within each of the layers approached along the Y-axis at a distance sufficient for the formation of covalent bonds with each other.

### 3.2. Energy Stability of Graphene/SWCNT Hybrid Films

**Figure 3:**
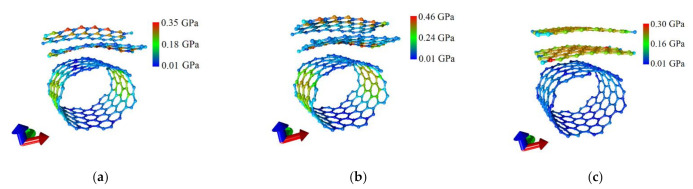
Distributions of local stresses of the atomic network of graphene/SWCNT hybrid films: (**a**) model V1; (**b**) model V2; (**c**) model V3.

As noted in Section 3.1, in the course of finding the equilibrium configuration of the constructed supercells of graphene/SWCNT hybrid films, both graphene nanoribbons and nanotube (12,6) were deformed. In this regard, it was necessary to assess the stability of graphene/SWCNT (12,6) structures to the arising deformations of the atomic network before proceeding to the study of their properties. Stability was estimated from the distribution of local stresses of the atomic network. The calculated distributions of local stresses for three supercells of graphene/SWCNT hybrid films are shown in Figure 3, where the gradation of stresses by atoms was shown in color. This figure shows that the distributions for the topological models V1 and V2 have a similar form: the highest stresses arise on the edge atoms of graphene nanoribbons, which were highly reactive due to the unsaturation of covalent bonds. The non-edge atoms of the nanoribbons have low stresses comparable to the stresses on the nanotube. The local stress distribution for the topological model V3 looks different. The highest stresses were experienced not by the edge atoms of the nanoribbons but by the atoms located in the central region of the nanoribbon. In this region, the convexity of the structure was observed and, consequently, the greatest curvature of the atomic network. This difference in the stress distribution over the atoms of the supercell can be explained as follows. In the case of the topological models V1 and V2, graphene retained the structure of nanoribbon with chemically active edge atoms during the formation of an extended fragment of graphene/SWCNT hybrid film by translation of a supercell. In the case of the topological model V3, graphene in an extended fragment of graphene/SWCNT hybrid film had a structure with linear dimensions differing from each other by less than three times. Therefore, the maximum stresses fell not on the edge atoms but on the sections of the atomic network with the maximum curvature. In this case, this was the central region of the structure with the maximum density of carbon atoms in the composition of the hybrid film. Turning to quantitative estimates of the stresses arising in the structure, it can be noted that the maximum values of stresses on atoms do not exceed ~0.46 GPa for all three considered topological models. Earlier, we found that the critical stress value for graphene structures was 1.8 GPa [47]. At such a stress value, the integrity of the atomic network begins to break. Consequently, the constructed models of supercells were highly resistant to arising deformations of the atomic network.

Another confirmation of the energy stability of the constructed supercells was the result of calculating the formation enthalpy Δ*H_f_*, which was determined by the following formula:

(3) $$\Delta H_{f}=\left(E_{film}-E_{gr}-E_{tube}\right)/N,$$

where *E_film_* is the energy of the hybrid film, *E_gr_* is the energy of graphene layers, *E_tube_* is the nanotube (12,6) energy, *N* is the number of atoms in the supercell. The structure of the hybrid film was configured in such a way that its total energy in absolute value was less than for its individual components. The Δ*H_f_* values are given in Table 1. It can be seen that each of the considered topological models was characterized by negative enthalpy, therefore, the final atomic configurations of supercells are energetically favorable.

### 3.3. Electronic and Electrically Conductive Properties of Graphene/SWCNT Hybrid Films

**Figure 4:**
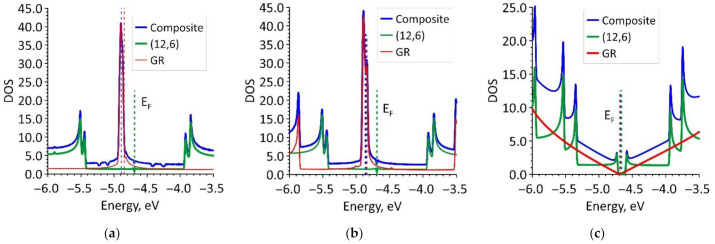
DOS of graphene/SWCNT hybrid films: (**a**) model V1; (**b**) model V2; (**c**) model V3.

The next stage of the study was to identify the features of the electronic structure of the studied graphene/SWCNT hybrid films. The distributions of the density of electronic states (DOS) were calculated for each of the constructed supercells. Our task was to determine how the DOS profile of graphene/SWCNT hybrid films was formed by evaluating the contribution of individual graphene nanoribbons and nanotube (12,6). Figure 4 shows fragments of the calculated DOS distributions of graphene/SWCNT hybrid films and their structural components for the energy range near the Fermi level *E_F_*. The values of *E_F_* for each topological model of the hybrid film are given in Table 2. We present this energy range for demonstration since the electronic states at the Fermi level make the decisive contribution to the conducting properties of a material. The plots in Figure 4 show how strongly the DOS profile at the Fermi level changes with a change in the width of the graphene nanoribbon. With a nanoribbon width of 2 (Figure 4a) and 3 (Figure 4b) hexagons, the DOS profile of graphene/SWCNT hybrid film at the Fermi level exhibited a high-intensity peak characteristic of graphene. This peak indicated the formation of localized states in the region of edge atoms of graphene nanoribbons. Consequently, it is graphene nanoribbons that determine the gapless nature of the band structure of graphene/SWCNT hybrid films with nanoribbon widths of 2 and 3 hexagons and, as a consequence, the metallic nature of their conductivity. In the case of a nanoribbon width of 4 hexagons (Figure 4c), the peak at the Fermi level completely disappears in the DOS profile of graphene/SWCNT hybrid film, but the energy gap *E_gap_* opens (see Table 2) as in the DOS of the nanotube (12,6). As noted above, with such a width of the nanoribbon, the translated supercell formed an extended 2D graphene/SWCNT structure, where graphene nanoribbons transformed into graphene sheets, for which the edges of the valence and conduction bands meet at a point at the Fermi level. Finally, as can be seen from Table 2, in this case, the position of the Fermi level changes from −4.86 eV to −4.68 eV, which corresponds to the Fermi level of an ordinary graphene sheet (−4.67 eV).

**Figure 5:**
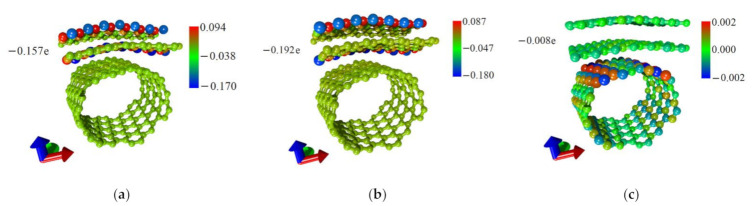
The electron charge density distribution over the atoms of the supercell of graphene/SWCNT hybrid films: (**a**) model V1; (**b**) model V2; (**c**) model V3.

To better understand the nature of the formation of DOS of graphene/SWCNT hybrid films, the distribution of the electron charge density over the atoms of the supercell was calculated. The calculated distributions are shown in Figure 5. As can be seen from Figure 5a,b, the pattern of charge distribution by the atoms of the upper and lower graphene nanoribbons for the topological models V1 and V2 coincides, which explains the similarity in the DOS profiles of these structures (Figure 4a,b). At the same time, in the case of model V2, a larger charge flowed from the nanotube to graphene (0.192*e* versus 0.157*e* for model V1), which led to an increase in the intensity of the DOS peak at the Fermi level for individual graphene nanoribbons in this case (Figure 4b). Now let us explain the significant difference in the distribution of the electron charge density of the model V3 (Figure 5c) in comparison with models V1 and V2. As described in Section 3.1, in extended fragments of graphene/SWCNT hybrid film constructed on the basis of models V1 (Figure 2a) and V2 (Figure 2b), graphene is represented as a chain of successive nanoribbons, each of which has chemically active edge atoms. Since the raised edge of one nanoribbon is located directly above the lowered edge of another nanoribbon, the edge atoms of neighboring nanoribbons will willingly exchange charge between themselves and with the atoms of the nanotube. In an extended fragment of graphene/SWCNT hybrid film constructed on the basis of the model V3 (Figure 2c), graphene is already represented not by a chain of nanoribbons, but by two sheets located one under the other with almost no displacement. Since bilayer graphene sheets are extended in two directions (X and Y), the influence of edge atoms on the electron charge density distribution of the graphene/SWCNT hybrid film will be minimal. Therefore, as can be seen from the data in Table 2, a small charge 0.008*e* transfers from the nanotube to the graphene in this case. The absence of charge transfer between graphene and a nanotube in the supercell of model V3 explains the minimum DOS at the Fermi level in Figure 4c.

**Figure 6:**
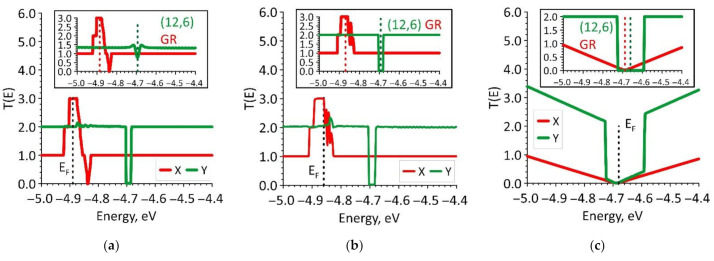
Plots of the transmission functions of graphene/SWCNT hybrid films (the inserts show the transmission functions for films of bilayer graphene nanoribbons and films of SWCNTs): (**a**) model V1; (**b**) model V2; (**c**) model V3.

After analyzing the electronic structure, we proceeded to study the nature of the electrical conductivity of graphene/SWCNT hybrid films. An important key to understanding this issue was to identify the features of electron transport. In this regard, we calculated the transmission function *T*(*E*), which characterized the probability of the passage of electrons through the given structure. The resulting plots of the function *T*(*E*) are shown in Figure 6 for the energy range near the Fermi level, since the *T*(*E*) profile near the Fermi level determines the value of electrical conductivity *G*. The *T*(*E*) plots for the direction of current transfer along the Y axis are marked in green, and along the X axis in red. The values of the *T*(*E*) in the plots are given in quanta of conductance *e*^2^/*h*. To explain the obtained dependencies, the *T*(*E*) plots of its constituent graphene and nanotube (in a square frame) are presented for each topological model of graphene/SWCNT hybrid film. The presented plots demonstrate that either graphene (current transfer in the direction of the X-axis) or a nanotube (12,6) (current transfer in the direction of the Y-axis) makes a decisive contribution to the formation of the *T*(*E*) profile. The *T*(*E*) profile contours of graphene/SWCNT hybrid film near the Fermi level largely repeat the DOS profile contours in this energy range. This allows us to use the conclusions about the influence of the electron charge density redistribution between the atoms of the nanotube and graphene layers, made in the analysis of the DOS, to explain the differences between the *T*(*E*) profiles of the models V1 and V2 and the *T*(*E*) profile of the model V3. In order to quantify the ability of graphene/SWCNT hybrid films to conduct current, we calculated the electrical resistances in the directions of current transfer along the X and Y axes (*R_x_* and *R_y_*, respectively). The resistance was calculated as the reciprocal of the electrical conductivity *G*, which was determined by Formula (2). The calculated resistance values are presented in Table 2. Analyzing the data in Table 2, it can be noted that the difference in resistance values for the topological models V1 and V2 in two directions of current transfer was not as noticeable. These resistances were about 5–6 kOhm, but *R_y_* was less than *R_x_*. This can be explained by the fact that the value of *T*(*E*) in the direction of the Y-axis is equal to “2” over a large energy interval, but in the direction of the X-axis, the *T*(*E*) reaches the value “3” at the Fermi level and then decreases to unity in almost the entire considered energy range. On the contrary, in the case of the topological model V3, the resistance values in different directions of current transfer differ by a factor of tens, i.e., the phenomenon of electrical conductivity anisotropy takes place. In the direction of the X-axis, the resistance reaches ~ 125 kΩ. This is explained by the fact that the function *T*(*E*) takes values less than unity over the entire energy range near the Fermi level. In the direction of the Y-axis, the resistance was about 16 kΩ, which was three times higher than the resistance values for models V1 and V2 since the *T*(*E*) at the Fermi level takes almost zero value in this case (see Figure 6c).

**Figure 7:**
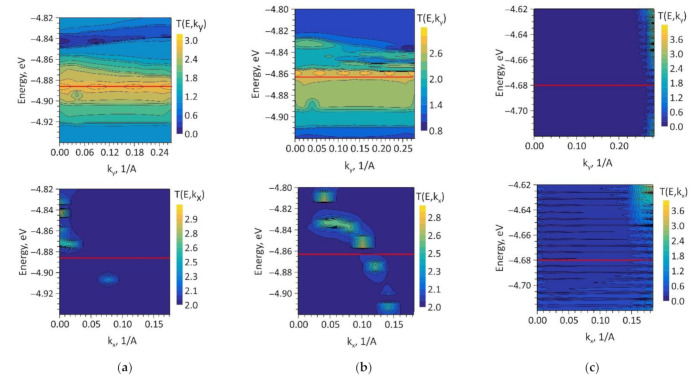
Maps of the transmission function in the coordinates of the energy *E* and wave number *k* of the Brillouin zone: (**a**) model V1; (**b**) model V2; (**c**) model V3.

For a visual interpretation of the *T*(*E*) profiles for the topological models V1, V2, and V3, we calculated the maps of the transmission function *T*(*k*, *E*), on which the wave number k was plotted. This wave number corresponds to the direction in which the partition of the reciprocal space was carried out. The calculated maps *T*(*k*, *E*) are shown in Figure 7. The red horizontal line indicates the Fermi level.

These maps clearly show that in the case of the current transfer in the direction of the X-axis (partition by *k_y_*), the function *T*(*E*) for the models V1 and V2 takes maximum values (of the order of 3), either at the Fermi level (model V1) or in the immediate vicinity of it (model V2). This result was in good agreement with the features of the profiles of the function *T*(*E*) in the vicinity of the Fermi level shown in Figure 6a,b. In the direction of the Y-axis (partition by *k_x_*), the function *T*(*E*) takes on the value “2” at almost all points of reciprocal space with the exception of individual “islands” explaining small deviations of the function *T*(*E*) from the value “2” near the Fermi level observed in Figure 6a,b. The function *T*(*E*) in the direction of the X-axis for the model V3 was zero throughout the reciprocal space, except for a small number of edge points located far from the Fermi level. As can be seen from Figure 6c, the function *T*(*E*) at the Fermi level and in its vicinity was close to zero and gradually increased with distance from it. In the direction of the Y-axis, the map *T(E,k)* was more rarefied since the function *T*(*E*) has a large number of conduction channels in this direction of current transfer. This explains the anisotropy of the conducting properties observed for this topological model of graphene/SWCNT hybrid film.

## 4. Conclusions

In this work, the theoretical and computational study of layered films formed by bilayer graphene and a layer of chiral SWCNTs was carried out. The results of this study made it possible to determine new topological models of graphene/SWCNT hybrid structures, characterized by high energy stability, and to reveal the regularities of the topological control of their electrically conductive properties. For the first time, at the level of computer simulation, we considered the atomic configurations of graphene/SWCNT hybrid films, in which the mutual arrangement of the graphene layers and the SWCNT layer led to the formation of structural regions with increased density, which corresponds to the data of modern experiments on the synthesis of graphene/CNT films [48]. Based on the results of calculating the DOS and the electron transmission functions, high sensitivity of the studied configurations of graphene/SWCNT hybrid films to the width of the graphene structure (zigzag nanoribbons) in the supercell was established. It was shown that at a nanoribbon width of 4 hexagons, the atomic structure of graphene/SWCNT hybrid film transforms from a configuration with numerous graphene nanoribbons arranged in the form of a “comb” at an angle to the nanotube surface to a configuration in which the nanotube is covered with two graphene sheets. This phase transition inevitably affects the ability of graphene/SWCNT hybrid film to conduct an electric current. We discovered the phenomenon of electrical conductivity anisotropy in such hybrid films with a nanoribbon width of 4 hexagons, which was absent for hybrid films with a nanoribbon width of 2 and 3 hexagons. The discovered phenomenon can form the basis for the operation of electronic devices such as a diode or field-effect transistor. The structures of graphene/SWCNT hybrid films with nanoribbon widths of 2 and 3 hexagons are promising objects for emission electronics. It is predicted that a ribbed or comb structure of graphene nanoribbons in the composition of graphene/SWCNT hybrid film can serve as a good electron nanoemitter during the operation of a field emission cathode.

## Figures and Tables

**Table 1:** Metric and energy characteristics of graphene/SWCNT hybrid films.

	Model V1	Model V2	Model V3
*L_x_*, nm	1.719	1.723	1.707
*L_y_*, nm	1.135	1.134	1.110
*r_x_*/*r_y_* (nanotube)	1.21	1.23	1.25
Δ*H_f_*, eV/atom	−0.17	−0.15	−0.14

**Table 2:** Electrophysical characteristics of graphene/SWCNT hybrid films.

	Model V1	Model V2	Model V3
*E_F_*, eV	−4.88	−4.86	−4.68
*E_gap_*, eV	0.000	0.000	0.001
Q on graphene, *e*	−0.157	−0.192	−0.008
*R_x_*, kOhm	7.53	6.22	125.89
*R_y_*, kOhm	6.58	5.95	16.31

## Data Availability

The data presented in this study are available on request from the corresponding author.
